# Prevalence of hearing loss in children following bacterial meningitis in a tertiary referral hospital

**DOI:** 10.1186/1756-0500-7-138

**Published:** 2014-03-11

**Authors:** Benson Wahome Karanja, Herbert Ouma Oburra, Peter Masinde, Dalton Wamalwa

**Affiliations:** 1 University of Nairobi, P.O. Box 2209-00202, KNH, Nairobi, Kenya; 2 Department Surgery, University of Nairobi, P. O. Box 30197-00100, GPO, Nairobi, Kenya; 3 ENT Department, Kenyatta National Hospital (KNH), University of Nairobi, P.O. Box 20723-00202, Nairobi, Kenya; 4 Department of Pediatrics and Child Health, University of Nairobi, P. O. Box 19676-00202, Nairobi, Kenya

**Keywords:** Hearing tests, Kenyatta national hospital, Sensorineural hearing loss (SNHL), Coma score, Seizures, Cranial nerve neuropathy, Positive CSF culture, Fever

## Abstract

**Background:**

This study aimed to examine hearing function in a group of children aged between the ages of six months and twelve years admitted with bacterial meningitis so as to determine the prevalence and degree of sensorineural hearing loss in them. This prospective study was conducted in the audiology unit and paediatric wards of Kenyatta National Hospital, KNH.

**Methods:**

The study involved 83 children (49 males and 34 females) between the ages of six months and twelve years admitted with bacterial meningitis. The median age for the children examined was 14 months (range from 5 to 120 months). They were sequentially recruited and at discharge following treatment, underwent age-appropriate hearing testing to evaluate presence and degree of hearing loss which was analyzed. The study was limited by the absence of otoacoustic emission and auditory brainstem responses testing by excluding the significant numbers of children below six months of age admitted with bacterial meningitis.

**Results:**

Thirty six of the 83 children (44.4%) were found to have at least a unilateral mild sensorineural hearing loss during initial audiologic testing. Of the children with hearing loss, 22 (26.5%) had mild or moderate sensorineural hearing loss and 14 (16.9%) had severe or profound sensorineural hearing loss.

**Conclusions:**

Sensorineural hearing loss was shown to be highly prevalent in children treated for bacterial meningitis. There is therefore a need for objective hearing assessment in infants and young children following bacterial meningitis and further studies involving larger population sizes.

## Background

Deafness is one of the commonest serious complications of bacterial meningitis in childhood. In developed countries, approximately 10% of survivors of bacterial meningitis are left with permanent sensorineural hearing loss [1-3]. Other children experience a transient hearing loss [3-6]. Both types of hearing impairment are thought to develop during the first few days of the illness [5-7].

Kenyatta National Hospital, KNH is Kenya’s national referral hospital. Estimates show that an average forty five children are admitted into its pediatric wards each month with a confirmed diagnosis of bacterial meningitis.

Behavioral tests of hearing may be used when an infant reaches the developmental age (as opposed to the chronological age) of six months. Infants not at this level of development and some of those with more than one disability will need to be tested by otoacoustic emissions (OAEs) and auditory brainstem responses (ABRs). Unfortunately the equipment for these latter two sets was unavailable forcing the study to be carried out in children above 6 months of age using behavioural of distraction testing.

So far, in KNH no similar study had been undertaken to determine the prevalence and burden of hearing loss following bacterial meningitis in children admitted to KNH.

## Methods

### Participants

The study involved 83 children (49 males and 34 females) between the ages of six months and twelve years admitted with bacterial meningitis from the pediatric wards, KNH. All cases admitted within twenty four hours of diagnosis, who met the inclusion criteria were recruited every weekday evening during the 3-month study period.

The lack of ABR and OAE testing limited the present study to behavioral tests of hearing which may only be used when an infant reaches the developmental age (as opposed to the chronological age) of six months.

All participants fulfilled the following criteria: age six months or older at the time of admission and confirmed diagnosis of bacterial meningitis. Bacterial meningitis was defined according to the World Health Organization (WHO) workbook recommendations based on laboratory findings, symptoms or signs [8]. Those excluded from the study included all with a confirmed diagnosis of or on current treatment for tuberculosis; prior history of hearing loss; using ototoxic antibiotics as part of treatment; chronic medical conditions (diabetes, renal, cardiac diseases); on treatment for malaria. Besides antibiotics all the patients received intravenous dexamethasone (0.6 mg/kg per day in four divided doses). The first dose of dexamethasone was administered 10-15 minutes before administration of antibiotic and was continued for four days.

A full medical history was documented and factors relating to the patient and prior treatment documented by the principal investigator in the questionnaire. The history included parental or a guardians’ assessment of hearing and any history of ear discharge or infection. Otoscopy was done using a Riester hand-held otoscope and its aural speculums and the findings recorded. The children then underwent a thorough physical examination. Hematological and CSF study results were documented. All these were entered in the patient’s proforma.

A full physical examination and hearing assessment was done prior to discharge from hospital and two weeks after. This identified and excluded transient hearing loss.

All participants’ parents or guardians gave informed consent for the study. Five guardians declined to have their children involved in the study. The study was approved by the institutional ethics and review committee of KNH.

#### Audiological protocol

All patients completed age-appropriate hearing tests carried out by trained audiologists in the ENT audiology section in a sound- proofed booth. This was well- lit with minimal littering to minimize the child’s distraction. The standard ambient noise was 35 dB. It was also well ventilated and large enough to accommodate the child and parent/ guardian, tester and distractor.

Children between six and twenty four months of age underwent the distraction test, those between twenty four and thirty six months of age had the performance tests done while pure tone audiometry was carried out on children above thirty six months of age. They were carried out in the following manner:

1 Behavioral testing

The behavioral testing equipment included toys that were not too bright used by the distractor in the behavioral tests to distract the child being examined. Three warblers were used: low, mid and high frequency types. A Manchester rattle was also used to deliver high frequency sounds during behavioral testing. The G-chime was used for mid-frequency sounds and the C-chime for high frequency sound generation.

a. Distraction Test

A distraction test was performed if the infant was sitting and able to turn and locate the source of a sound. It was carried out with the infant sat upon an adult’s knee facing forwards where a distractor controlled the infant’s attention using toys. The tester introduced the sound signals at high, mid and moderate frequencies from 45 degrees and one meter behind, at the level of the ear. These were tested separately in order to detect hearing loss restricted to one part of the frequency range. The sounds were introduced at very quiet levels (35dBA). Care was taken not to give clues as to the tester’s position other than the test signal.

a. Performance Tests

The child was shown how to wait until a sound was heard before carrying out an action. Once this could be done the test was be performed at a meter distance and from behind. The test was performed using “Go” for low frequencies, “S” for high frequencies, introduced at the quietest voice levels or **2** Warble tones at 500 Hz, 1or 2 kHz and 4 kHz introduced at a very quiet level corresponding to normal hearing. The child was said to have “passed” the screen if there were two responses at the quietest level.

2 Pure Tone Audiometry

This was carried out in all children recruited for the study above thirty six months of age. Pure tones (20dDHearing Level, HL) were introduced using headphones and testing carried out by air conduction (500 H-4000 Hz) and bone conduction (500-4000 Hz). Pure tone audiometry was performed using an Interacoustics clinical audiometer (Model AC33; serial no. SN735530, calibration date June 2009). Sound delivery and masking during pure tone audiometry was ensured using TDH-399 headphones. No sponsorships or competing interests were involved in these tools.

The main outcome measure was presence or absence of sensorineural hearing loss in the children.

### Follow up

If the child had made a complete recovery from meningitis, lived far from the hospital, and had no hearing loss, follow up was not done.

If the child had sequalae that required further management beyond 2 weeks, follow up was continued. For those children exhibiting hearing loss, whether conductive or sensorineural, recommendations for management and follow-up were based on specific test findings and varied accordingly.

### Statistical analysis

Descriptive statistics (means for continuous variables and proportions for categorical variables) were calculated to describe the population.

The main outcome was degree of sensorineural hearing loss as measured by the age-appropriate hearing test. This had three levels of outcomes: normal, mild/moderate and severe/profound. The population proportions and the 95% confidence interval, CI were estimated for each category of outcome, using statistical methods to give more precise estimates. All categorical variables were cross tabulated with the outcome and Pearson Chi Square computed.

## Results

**Figure 1:**
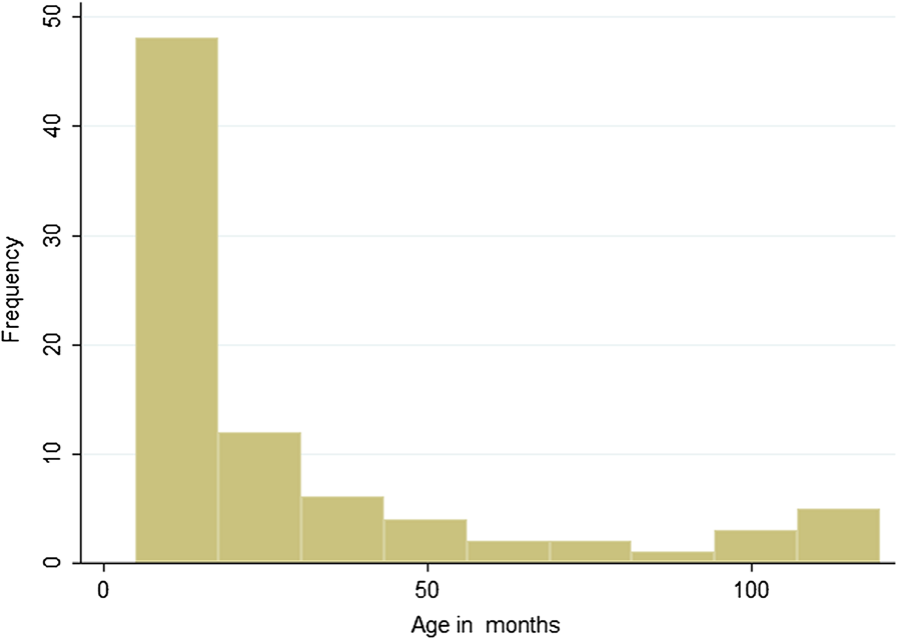
Age distribution in months of children evaluated.

We analyzed the outcomes of eighty three children (49 males and 34 females) admitted with bacterial meningitis during the three month study period. The median age for the children examined was 14 months (range from 5 to 120 months). This is represented along with the mean on Table 1. The characteristics of the children studied are presented in Figure 1.

**Table 1:** Mean and Median Age Ranges

**mean (SD)**	**Median (range)**
29.09(31.71)	14(5-120)

A significant number of the patients enrolled developed sensorineural hearing loss as a sequalae (36/83). The overall prevalence of SNHL loss was estimated to be 43.37% (95% CI: 33.22, 55.93). Of the children with hearing loss, 22 (26.5%) had mild or moderate sensorineural hearing loss, and 14 (16.8%) had severe or profound sensorineural hearing loss. The prevalence of specific categories of SNHL is presented in Table 2.

**Table 2:** Prevalence of SNHL

**SHNL category**	**Prevalence (%)**	*Normal based 95%CI
Normal	47/83 (56.62)	[44.79, 66.05]
Mild/moderate	22/83 (26.51)	[17.33, 35.68]
Severe/profound	14/83 (16.87)	[10.22, 25.93]
Overall SNHL	36/83 (43.37)	[33.22, 55.93]

Only seventeen (20.5%) of CSF specimens examined cultured any bacteria. *Streptococcus pneumoniae, Haemophilus influenzae* and *Neisseria meningitidis* were isolated in 10, 4, and 3 children respectively. There were no distinctly different clinical presentations among children with *S pneumoniae, H influenzae* and *N meningitidis* meningitis.

## Discussion

In the present study children proven by history and CSF findings to have bacterial meningitis were evaluated and found to have a sensorineural hearing loss prevalence following the initial hospitalization of 43.4% (95% CI). This was therefore greater than findings of previous studies. Walter et al reported an incidence of 14% [9] consistent with other reports [2,4,10,11]. This is likely due to several factors. The study institution is a tertiary referral center for a major metropolitan area that understandably receives a disproportionate number of very sick children. In addition, perhaps current pathogens are more virulent owing to continued drug-resistance. Finally, most children in this study had not had previous objective audiologic testing, and a negative history for hearing loss was based on history alone. Therefore, a few of them may have had a previously undiagnosed hearing loss despite attempts at identifying that by the principal investigator using the questionnaire. This could potentially inflate the prevalence of hearing loss; however, it is unlikely this would significantly affect the overall prevalence.

Consistent with most prior studies, this work did not reveal any relationship between occurrence and severity of hearing loss to the male gender. Forty nine (59%) of the children evaluated were male while 34 (41%) were female. There were no differences in the prevalence of hearing loss between the two groups. Walter et al. showed that being male was a significant independent risk factor for hearing loss [9]. Early age at illness was identified by Grimwood et al as a significant risk factor for hearing loss, with children suffering meningitis before twelve months of age performing more poorly than children suffering meningitis later in infancy and childhood, and age-matched controls, on measures of language and reading skills [12]. However, we found that neither age nor sex affected hearing outcome. This is in agreement with most previously reported studies [8,13-16].

## Conclusions

Hearing loss is highly prevalent in children treated for bacterial meningitis in Kenyatta National Hospital with a prevalence of 43.4%. Age at illness was found not to be a significant determinant of hearing loss following bacterial meningitis. There exists a need for objective hearing assessment in infants and young children following bacterial meningitis. This should be mandatory in all patients treated for bacterial meningitis.

## Competing interests

No sponsorships or competing interests have been disclosed for this article.

## Authors’ contributions

Conceived and designed the experiments: BWK. Analysed the data: BWK. Wrote the first draft of the manuscript: BWK. Contributed to the writing of the manuscript: HOO, DW. Agree with manuscript results and conclusions: HOO, DW, PM. Jointly developed the structure and arguments for the paper: BWK, HOO, PM. Made critical revisions and approved final version: HOO, DW. All authors reviewed and approved of the final manuscript.
